# Electrical Double-Layer Transistors Comprising Block Copolymer Electrolytes for Low-Power-Consumption Photodetectors

**DOI:** 10.1021/acsami.4c01959

**Published:** 2024-05-06

**Authors:** Hung-An Lin, Yi-Hsun Weng, Tiffany Mulia, Cheng-Liang Liu, Yan-Cheng Lin, Yang-Yen Yu, Wen-Chang Chen

**Affiliations:** † Department of Materials Engineering, Ming Chi University of Technology, New Taipei City 24301, Taiwan; ‡ Department of Chemical Engineering, National Taiwan University, Taipei 10617, Taiwan; § Advanced Research Center for Green Materials Science and Technology, National Taiwan University, Taipei 10617, Taiwan; ∥ Department of Materials Science and Engineering, National Taiwan University, Taipei 10617, Taiwan; ⊥ Department of Chemical Engineering, National Cheng Kung University, Tainan 70101, Taiwan

**Keywords:** electrical double layers, phototransistors, block copolymers, polyelectrolytes, photodetectivity

## Abstract

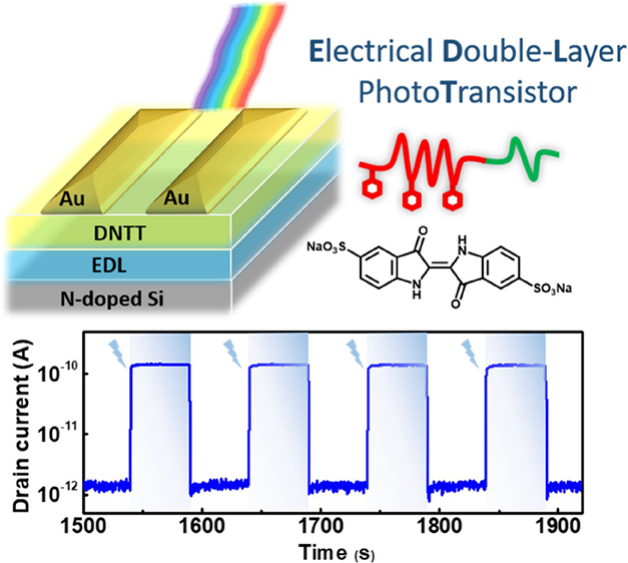

Electrical double-layer transistors (EDLTs) have received extensive research attention owing to their exciting advantages of low working voltage, high biocompatibility, and sensitive interfacial properties in ultrasensitive portable sensing applications. Therefore, it is of great interest to reduce photodetectors’ operating voltage and power consumption by utilizing photo-EDLT. In this study, a series of block copolymers (BCPs) of poly(4-vinylpyridine)-*block*-poly(ethylene oxide) (P4VP-*b*-PEO) with different compositions were applied to formulate polyelectrolyte with indigo carmine salt in EDLT. Accordingly, PEO conduces ion conduction in the BCP electrolyte and enhances the carrier transport capability in the semiconducting channel; P4VP boosts the photocurrent by providing charge-trapping sites during light illumination. In addition, the severe aggregation of PEO is mitigated by forming a BCP structure with P4VP, enhancing the stability and photoresponse of the photo-EDLT. By optimizing the BCP composition, EDLT comprising P4VP_16k_-*b*-PEO_5k_ and indigo carmine provides the highest specific detectivity of 2.1 × 10^7^ Jones, along with ultralow power consumptions of 0.59 nW under 450 nm light illumination and 0.32 pW under dark state. The results indicate that photo-EDLT comprising the BCP electrolyte is a practical approach to reducing phototransistors’ operating voltage and power consumption.

## Introduction

In this modern era, the use of electronic devices significantly increases and boosts the demand for performance improvement. To obtain suitable devices, low working voltage and power consumption are required for electronic and biomedical necessities. The electrical double-layer (EDL) capacitor indicates the movement of cations and anions at the interface between an electrode and an electrolyte in a liquid or solid state with an external electric field applied between the electrode and electrolyte.^1−4^ Accordingly, electrical double-layer transistors (EDLTs) are introduced because they can work at a low working voltage, which is favorable for achieving such functionality. In EDLTs, the carrier density of the semiconductor channel can be modulated by an ion-induced EDL capacitance at the interface between the semiconductor layer and electrolyte.^5−8^ The EDL capacitance can control the flow of charge carriers in the semiconductor channel by applying a gate voltage to the electrolyte, modifying the device’s electrical characteristics. Not only are they able to work at low voltage operation, but EDLTs have also received extensive attention owing to their advantages of high biocompatibility and sensitive interfacial properties in ultrasensitive portable sensing applications. Diverse EDLT designs have been explored, including organic-based,^9^ nanomaterial-based,^10^ oxide-based,^11,12^ and neuromorphic EDLTs^6^ approaches. For instance, Zheng et al. reported a planar flexible floating-gate EDLT that included the excellent ability of low threshold voltage, high transconductance, and stable ion-concentration-dependent switching capability. The flexible device exhibited promising memory performance at different bending angles, indicating good flexibility and mechanical stability of the EDLT devices.^13^

Organic phototransistors have been referred to as exceptional optoelectronic devices owing to their light weight, low cost, compatibility with integrated circuits, and ability to detect light and amplify signals.^14−17^ Various ideas have been incorporated into phototransistors to improve device performance or endow memory capability, including floating gates^18−22^ and polymer electrets.^23−30^ In phototransistors, the combination of light and the external gate voltage is applied to modulate the carrier concentration in the semiconducting channel. Therefore, the gate voltage and light can configure the channel conductivity, allowing for highly tunable device performance. In addition, photoactive materials or polymer electrets are commonly used in phototransistor memory to store charges generated by light absorption. With a judicious design in device operations, memory devices based on polymer electrets can be electrically and optically programmed and erased. Hence, phototransistors are proven to be excellent nonvolatile memory devices, as they store light signals even after power turns off. Thus, they are regarded as promising candidates for high-performance photomemory with multilevel and rapid photoresponse.

Light utilization for information transmission has been increasingly required with the widespread adoption of wireless and mobile communication. Beyond the application of electrical transistors and memory, EDLT can be further applied in photonic devices. Recently, Sun et al. introduced a near-infrared organic phototransistor at low working voltages with an electrolyte gate. In addition to its benefit of reducing operating voltage, the electrolyte gate is lightweight, flexible, low-cost, biocompatible, and environmentally friendly. Notably, persistent photoconductivity that can be used for photomemory was observed even when the device was exposed to air in this photo-EDLT memory.^31^ Nonetheless, there are still few reports about the application of EDLT for a photodetector. To fully utilize the merits of EDLT, including the low operating voltage and power consumption, in this work, a series of poly(4-vinylpyridine)-*block*-poly(ethylene oxide) (P4VP-*b*-PEO) with different molecular weights and compositions were applied to formulate polyelectrolyte for photo-EDLT. The block copolymers (BCPs) studied are P4VP_16k_-*b*-PEO_5k_ (P3E1), P4VP_17k_-*b*-PEO_17k_ (P1E1), and P4VP_4.5k_-*b*-PEO_32k_ (P1E7). Dinaphtol[2,3-*b*:2′,3′-*f*]thieno[3,2-*b*]thiophene (DNTT) was chosen as the semiconducting layer, and the corresponding BCPs were blended with indigo carmine salt acting as the ionic species and the photogate inside the BCP electrolyte. PEO was used as the ionic conducting matrix to form an EDL phenomenon between the electrodes under an applied electric field. The severe aggregation of PEO is expected to be mitigated by adopting a BCP structure with P4VP, enhancing the stability and photoresponse of photo-EDLT. The optical and morphological properties of the BCP electrolyte films were studied by using UV–vis optical absorption, photoluminescence (PL) emission spectroscopy, and atomic force microscopy (AFM). The photodetecting capability of the photo-EDLT devices comprising a series of BCP electrolytes was investigated to corroborate their structure–performance relationship.

## Experimental Section

### Materials

Block copolymers of P4VP_20k_-*b*-PEO_5k_ (P4E1), P4VP_16k_-*b*-PEO_5k_ (P3E1), P4VP_17k_-*b*-PEO_17k_ (P1E1), and P4VP_4.5k_-*b*-PEO_32k_ (P1E7) were ordered from Polymer Source. Poly(4-vinylpyridine) (P4VP, Mw. = 160,000 g/mol), poly(ethylene oxide) (PEO, Mw = 100 000 g/mol), and *N*, *N*-dimethylformamide (DMF, anhydrous, 99.8%) were purchased from Sigma-Aldrich. Dinaphtho[2,3-b:2′,3′-f]thieno[3,2-*b*]thiophene (DNTT, 99%) was purchased from Luminescence Technology Corp.

### Fabrication of the Electrical Double-Layer Transistors

To prepare the BCP electrolyte films, BCPs were dissolved in DMF at a concentration of 40 mg mL^–1^, and indigo carmine was added to the solutions at a concentration of 20 mg mL^–1^. The solution was stirred overnight at 60 °C and filtered through a polytetrafluoroethylene membrane syringe filter with a pore size of 0.22 μm to remove the residual aggregates. Next, the BCP solution was spin-coated onto a highly *n*-doped silicon wafer at 650 rpm for 60 s, followed by 1300 rpm for 15 s. The BCP electrolyte films were annealed at 60 °C under vacuum for 1 h, and the film thickness spanned the range of 200–300 nm. Note that the wafer substrate was precleaned by using a UV-ozone cleaner before use. Subsequently, a 60 nm-thick DNTT layer was thermally deposited at 10^–7^ Torr onto the BCP electrolyte film, and the Au electrodes with a thickness of 40 nm were deposited at 10^–7^ Torr through a patterned shallow mask with the channel length (*L*) and width (*W*) of 1000 and 50 μm, respectively.

### Characterization

The UV–vis absorption and PL spectra of the BCP electrolyte films were measured by using a Hitachi U-4100 spectrophotometer and a Horiba Jobin Yvon Fluorolog-3 spectrofluorometer. A cyclic voltammetry (CV) profile of indigo carmine and BCP films was conducted by using a CHI 6273E electrochemical analyzer using a three-electrode cell system, where Ag/AgCl and Pt rod were used as a reference and an auxiliary electrode, respectively, and the electrolyte was composed of 0.1 M tetrabutylammonium perchlorate in anhydrous acetonitrile. The film thickness of the BCP electrolytes was measured using an optical thickness meter (OPTM-A3, Otsuka Electronics Co., Ltd.). The surface morphology of BCP electrolyte films was investigated using an AFM instrument (Bruker Innova) operating in tapping mode. The hydrophilicity of the BCP electrolyte films was evaluated by using a contact angle analyzer (CAM120, Creating Nano Technologies, Inc.) to capture the pattern of the water droplets on the films. The Fourier transform infrared spectroscopy (FTIR) spectra were analyzed by a PerkinElmer Spectrum Two FT-IR L160000F. The morphology of DNTT was obtained by using scanning electron microscopy (SEM, JEOL JSM-7600F) and grazing-incidence wide-angle X-ray scattering (GIXD) at BL13A1, National Synchrotron Radiation Research Center, Taiwan. The incidence angle of the X-ray was set as 0.12°. Energy-dispersive X-ray (EDX) mapping was conducted by using a JEOL JSM-5310 equipped with a Link eXL EDX system to analyze the elemental composition of BCP electrolytes. The time-resolved photoluminescence (TRPL) profile was characterized by using a HORIBA Jobin Yvon Fluorolog-3 spectrofluorometer with 365 nm laser excitation (DD-375L, HORIBA) and an analyzer (DeltaDiode, HORIBA). The TR-PL profile was fitted with a biexponential function. The electrical characterizations, including capacitance measurement and transfer characteristics, were conducted using a Keithley 4200-SCS semiconductor parameter analyzer equipped with a digital capacitance–voltage measurement unit in a nitrogen-filled glovebox. The areal capacitance (*C*_areal_) of the BCP electrolyte films in the form of metal–insulator–metal (MIM) structure was measured at the range of 1 kHz to 1 MHz. The hole mobility (μ_h_) was estimated from the slope in the saturation regime of the transfer curve in the form of the square root of source-to-drain current (*I*_ds_^1/2^) versus the gate voltage (*V*_g_) using eq 1:
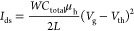
1

The transfer curves of EDLTs were measured under *V*_d_ = −1 V and *V*_g_ sweeping from 1 to −3 V. Transient photocurrent characteristics of the photo-EDLTs were measured by using Keithley 2634B in a nitrogen-filled glovebox. A 450 nm laser generated by a monochromatic photonic source (Sadhu Design Co.) with an adjustable light intensity spanning the range of 0.27–189 mW cm^–2^ was applied to illuminate the EDLT. Note that the light intensity was calibrated by using a laser power meter (Thorlabs PM 100D).

## Results and Discussion

### Optical and Electrochemical Characterizations of the BCP Electrolyte Films

**Figure 1:**
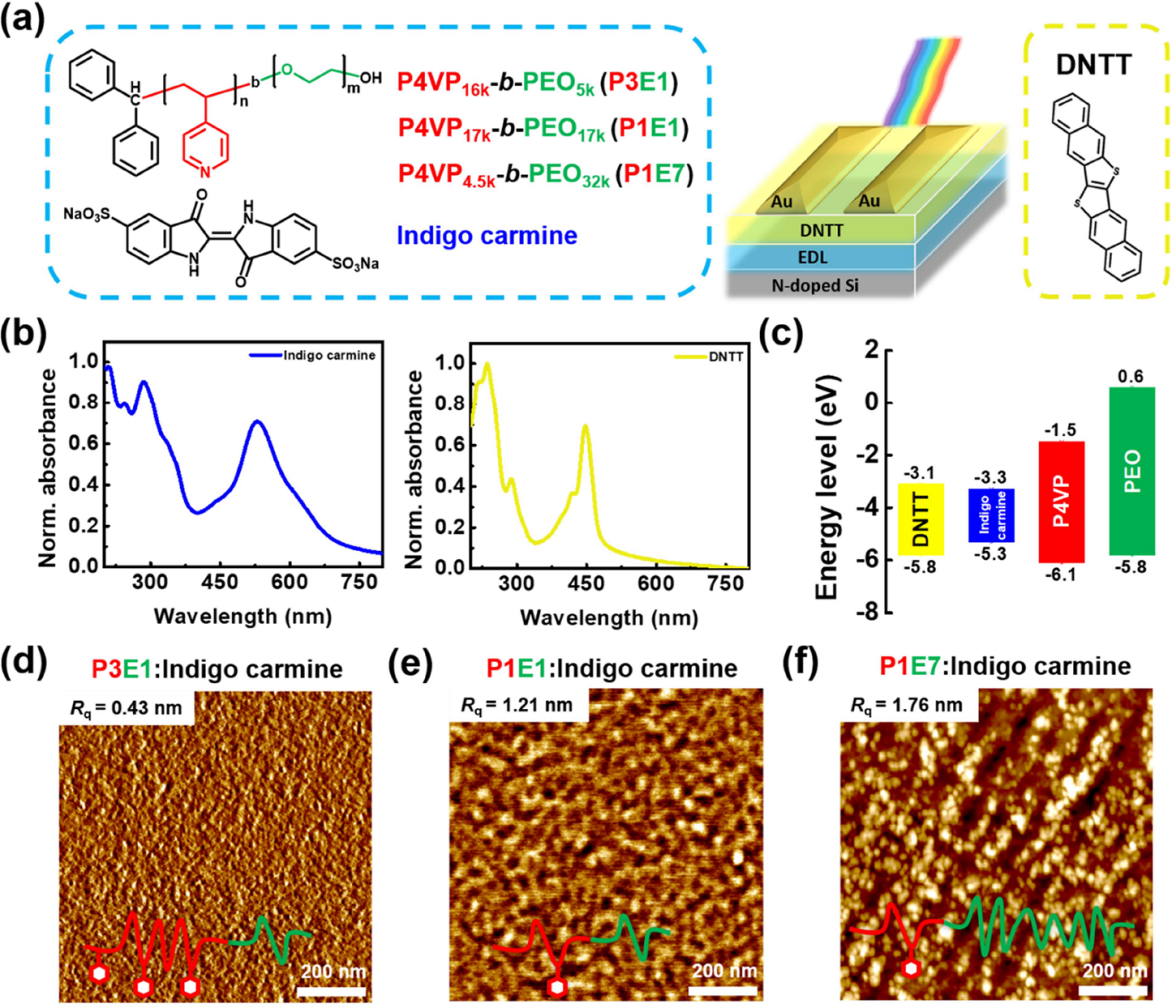
(a) Chemical structures of the constituent materials in the BCP electrolytes and the semiconducting channel for photo-EDLT. (b) UV–vis absorption spectra of indigo carmine (left) and DNTT (right). (c) Energy levels of the constituent materials in EDLT. AFM topographies of the BCP electrolyte films comprising (d) P3E1, (e) P1E1, and (f) P1E7 with indigo carmine.

To develop EDLT for photodetectors, a series of BCPs comprising P4VP and PEO with varied compositions were applied. In this study, indigo carmine, a light-absorbing salt, was blended with the BCP to form a polyelectrolyte and provide a photoresponse to the devices. PEO is a highly ion-conductive polymer with a high crystallinity. Hence, the BCP structure was employed to reduce the crystallinity of PEO and foster microphase separation.^32−34^ Additionally, P4VP was selected as the other block due to the existence of weak lone-pair interaction of pyridine with ions and the charge-trapping capability in field-effect transistors, making it suitable to combine with PEO to perform the EDLT.^35,36^Figure 1a illustrates the device architecture of the EDLT devices studied. The devices were prepared in a bottom-gate/top-contact (BG/TC) configuration with the channel material of DNTT coated above the EDL layer, and the EDL layer consisted of the BCP electrolyte with indigo carmine. The optical properties of these constituent materials are presented in Figures 1b and S1, displaying the UV–vis absorption and PL emission spectra of indigo carmine and DNTT. Based on the UV–vis analysis, indigo carmine absorbs green light from 500 to 550 nm, while DNTT absorbs blue light in the 400–450 nm range. Moreover, indigo carmine and DNTT showed adjacent light emission under excitation at 260 nm, in the ranges of 350–450 and 450–550 nm, respectively. In comparison to DNTT, indigo exhibits a significant Stokes shift.^37^ This can be correlated to the less tightly packed structure and ionic functional groups in indigo carmine, leading to a better distribution within the polar medium. On the other hand, DNTT forms a tightly packed molecular crystal that causes a slight Stokes shift and apparent overlapping absorption and emission spectra.

Figure 1c displays the energy levels of the materials studied. The highest occupied molecular orbital (HOMO) levels were determined from CV analysis. Figure S2 demonstrates the oxidation peaks of the materials, and the first onset potential was used to calculate the HOMOs. Accordingly, the HOMO levels of DNTT, indigo carmine, P4VP, and PEO are −5.8, −5.3, −6.1, and −5.8 eV, respectively. The optical bandgap (*E*_g_) was estimated from the Tauc plots of the materials studied (Figure S3). The lowest unoccupied molecular orbital (LUMO) levels were calculated by the equation LUMO = HOMO + *E*_g_. Therefore, the estimated LUMO levels of DNTT, indigo carmine, P4VP, and PEO are −3.1, – 3.3, – 1.5, and 0.6 eV, respectively. Due to the large bandgap of P4VP and PEO, the BCPs can act as insulating matrices. In addition to the CV profile of indigo carmine, the electrochemical properties of BCP films were also investigated. The HOMO and LUMO levels are (−6.0, −1.4) eV for P3E1, (−6.2, −1.6) eV for P1E1, and (−6.2, −1.6) eV for P3E1. The results warranted the electrochemical stability of the selected BCPs under a wide range of potential windows, which can be attributed to the decent oxidative and reductive stability of the constituent homopolymers of PEO and P4VP. Based on the optical and electrochemical properties, indigo carmine and DNTT are suitable for utilization in EDLT as a photogate and a channel material, and the BCPs are favorable for the EDL layer.

### Morphological Characterization of the BCP Electrolyte Films

In this PEO-*b*-P4VP series, the amount of P4VP and PEO in the BCPs determines which block acts as the matrix and domain because of the significant difference in the molecular weight. To quantify the proportions of each BCP, the volume fractions were calculated by using the reported density of PEO (1.2 g cm^–3^) and P4VP (1.15 g cm^–3^). The volume fractions of the PEO block (*f*_PEO_) in P3E1, P1E1, and P1E7 are 0.11, 0.27, and 0.95, respectively. In P1E7, the molecular weight of PEO is significantly larger than that of P4VP, leading to higher *f*_PEO_ among the studied BCPs. As a result, PEO acts as the background material (matrix) surrounding the distributed P4VP domain. In contrast, P4VP serves as the matrix instead in P3E1 and P1E1 due to the larger amount of polymer in the BCPs (*f*_P4VP_ = 0.89 and 0.73). However, the crystallinity of PEO is so high that it greatly influences the whole morphologies of the BCPs. The hydrophilic nature of PEO results in the polymer tending to move toward the environment, leading the P4VP to assemble while encircled by the PEO block. As can be seen in Figure S4, the water contact angles of these polyelectrolyte films are 70.5°, 43.1°, 40.4°, 20.2°, and 10.5° for P4VP, P3E1, P1E1, P1E7, and PEO, respectively. This trend is in good accordance with the distribution of P4VP and PEO blocks in the BCP electrolyte films; that is, the BCPs with higher content of PEO exhibit lower water contact angles owing to their high polarity. Surprisingly, the water contact angle of P3E1 and P1E1 is considerably lower compared to that of P4VP, despite having a greater proportion of P4VP in the BCPs. This is attributed to the PEO block in the BCP that is prone to exposure to the outer environment, in contrast to P4VP. Moreover, the addition of salt increases the volume fraction of the PEO block, as indigo carmine prefers to interact with PEO over P4VP. Hence, the PEO block acts as the matrix in P3E1 and P1E1, elucidating the lower water contact angle in these respective BCPs.

To gain insight into the surface morphology of the BCP electrolyte films, the surface nanostructures were observed by using AFM, as presented in Figures 1d–f and S5. As can be seen from the AFM topographies, the surface roughness (*R*_q_) decreased as the amount of P4VP in the BCPs increased. This phenomenon can be ascribed to the highly crystalline PEO atom within the BCPs. As depicted in Figure S5b–d, the BCPs tend to form phase separation, resulting in disorder nanostructures because of the immiscibility between PEO and P4VP, not to mention the high *R*_q_ of 4.41 and 2.69 nm due to the abundant PEO segment in P1E1 and P1E7, respectively. Among the BCP pristine films, P1E7 (Figure S5d) exhibits severe phase separation and adopts a fiber-like morphology similar to that of PEO pristine film (Figure S5e). Nevertheless, the AFM images in Figure 1d–f show granular nanostructures and decreased *R*_q_ values after the addition of indigo salt. These results indicate that the immiscibility of the two blocks can be reduced by disrupting the crystalline structure of PEO with the introduction of indigo carmine. In particular, indigo carmine can be well dispersed in the P1E1 (Figure 1d) and P3E1 (Figure 1e) polymer layers owing to the lower content of PEO in the BCPs. Conversely, Figure 1f demonstrates that P1E7 maintains a high *R*_q_ (1.76 nm) even with the presence of indigo salt. Accordingly, the highly crystalline PEO is not conducive to the dispersion of indigo carmine, resulting in severe aggregation in the thin films. In addition to the crystal formation of PEO, Bronstein et al. reported that PEO-*b*-P4VP tends to form a micelle structure with PEO surrounding the P4VP core.^38^ This phenomenon is also observed from PEO-*b*-P2VP, which was also reported by the same group.^39^ Accordingly, the BCPs with higher PEO contents are prone to coalescing into small and broadly distributed micelles to encapsulate metal nanoparticles. This phenomenon can help explain the AFM morphology in this study. The high roughness and aggregates of P1E7 may be attributed to the BCP micelles, and some of the salt may be confined inside the micelle, which is not beneficial for device performance. Moreover, we investigated the surface conditions of the blend films comprising PEO, P4VP, and indigo carmine. For ease of comparison, we set the blending weight ratio the same as the block ratio of the BCPs. As can be seen from Figure S6, all the AFM topographies exhibit severe aggregation and high surface roughness. These outcomes strongly suggest that BCP formation is helpful for the distribution of the photogate, which can be significantly beneficial for device operation.

AFM can provide only a rough evaluation of the surface patterns. To gain further insight into the self-assembly of BCP electrolyte films, grazing-incidence small-angle X-ray scattering (GISAXS) was conducted to analyze their domain size. As can be seen in Figure S7, a strong diffraction at *q*_y_ = 0.034 Å^–1^ can be observed from P3E1; the corresponding domain size is 18.5 nm. A weak diffraction at *q*_y_ = 0.053 Å^–1^ can be observed from P1E7; the corresponding domain size is 11.8 nm. In comparison, there is no observable diffraction in P1E1’s pattern. Therefore, the BCP electrolyte with a low PEO content is beneficial for salt accommodation and self-assembly.

To investigate the dispersion of indigo carmine salt in the BCP matrix, we analyzed the SEM images and EDX elemental mapping of our BCP composite films. Figure S8 shows the SEM images of the BCP composite films, and Figure S9 shows the EDX mapping of the sodium, sulfur, and nitrogen elements. From the distribution of sulfur, we can observe that indigo carmine is more uniformly dispersed in P3E1. However, in P1E1 and P1E7, there are increasing blank spaces within the mapping. From the distribution of nitrogen, we observe that the blank spaces are approximately located in the same place as those in the sulfur distribution. Therefore, we deduct that the blank spaces are the crystalline region of PEO, and indigo carmine should inhabit P4VP or amorphous PEO, consistent with our previous discussions. On the other hand, sodium is uniformly distributed in all three BCP composites, which is due to the dissociation of indigo carmine. Therefore, the BCP with a lower PEO content is beneficial for a homogeneous allocation of indigo carmine in the thin film, and the block ratio of P3E1 is sufficient for dissociating the salt. To understand the interactions between indigo carmine and the parent polymers, we conducted Fourier transform infrared spectroscopy (FTIR) of the BCP composite films. The samples were prepared by drop casting the solutions on KBr pellets and were dried under a vacuum afterward. Figure S10 shows the FTIR spectra of the BCP composites. The lines around 1598 and 993 cm^–1^ imply the stretching mode of pyridine, and the line located around 1101 cm^–1^ represents the stretching mode of C–O–C.^40,41^ The peak shifting around 1598 cm^–1^ increases as the blocking ratio of P4VP increases, indicating improved interactions between the P4VP segment and indigo carmine. However, we cannot observe a similar trend in the C–O–C stretching mode. It is due to the coexistence of PEO amorphous and crystalline regions, while indigo carmine prefers to blend in the amorphous region. In addition, as can be seen in Figure S11, the blends of P4VP or PEO with indigo carmine showcase poor homogeneity. The salt cannot be properly accommodated inside the homopolymer matrix. This comparison highlights the importance of a good BCP design in improving the morphology of polyelectrolyte systems.

### EDLT Characterization

**Figure 2:**
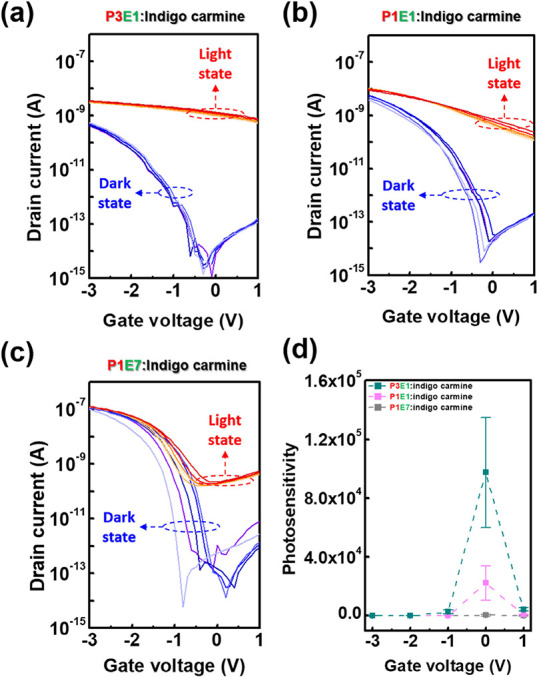
Transfer characteristics of the EDLT comprising (a) P3E1, (b) P1E1, and (c) P1E7 with indigo carmine in the dark state or under 450 nm light illumination. (d) Sensitivity of the EDLT with various BCP electrolytes. Note that the gate voltage applied was swept from 1 to −3 V, the drain voltage was fixed at −1 V, and the light intensity was 155 mW cm^–2^. The measurements were conducted in quintuplicates, and the sensitivity was averaged from them.

To investigate the structure–performance relationship of the BCP electrolytes in EDLTs, the devices were fabricated by spin-coating the BCP:indigo carmine solution on a precleaned bare wafer, subsequently followed by depositing DNTT and gold electrodes to obtain the EDLT with a BG/TC device configuration. The details of device fabrication and characterization can be seen in the Experimental Section. Figures 2a–c and S12 depict the repeated transfer characteristics and hysteresis of EDLTs comprising BCP electrolytes of P3E1, P1E1, and P1E7, respectively. The transfer characteristics of reference devices comprising P4VP or PEO with indigo carmine are presented in Figure S13. The gate and drain voltages were set from 1 to −3 and −1 V for characterizing the *p*-type transistors. In the dark state, all the EDLTs demonstrated clear depletion and accumulation regions, and the inversion was due to the negative gate bias that facilitated indigo carmine anions to accumulate at the semiconductor/EDL interface and induced the positive hole carriers. We can observe a clockwise hysteresis on both the dark state and 450 nm illumination of all the EDLTs, and the hysteresis dissipates soon after a round of sampling. Therefore, the hysteresis may have originated from the ion traps triggered by the electric field. When the electric field under the measurement is turned off, the ions are allowed to detrap under relaxation. To evaluate the hole mobility (μ_h_) of these EDLTs, the areal capacitance (*C*_areal_) of the BCP electrolytes was measured in advance. As demonstrated in Figure S14a**,** P1E7 exhibited the highest *C*_areal_ of 295.9 ± 11.1 nF cm^–2^, while P1E1 and P3E1 showed lower *C*_areal_ values of 60.3 ± 2.0 and 28.2 ± 3.3 nF cm^–2^, respectively. This phenomenon is related to immobile indigo carmine in the P4VP matrix that fails to form the EDL effect under voltage bias. Figure S14b depicts the areal capacitance–frequency relations of the three EDLs. Most polymer electrolytes show declining capacitance above 1 kHz frequency due to the lower ion mobility in solid electrolytes.^42−44^ Therefore, the curves showed decreasing *C*_areal_ as the frequency increased because dipolar relaxation becomes dominant at high frequencies.

The μ_h_ of EDLTs was calculated and is summarized in Table 1. As can be seen, the μ_h_ values of P3E1, P1E1, and P1E7 are 6.76 × 10^–4^, 2.61 × 10^–3^, and 7.15 × 10^–3^ cm^2^ V^–1^ s^–1^, respectively. The disparity in device current can be attributed to the EDL capacitance and the distribution of P4VP/PEO blocks at the contact interface with DNTT. In addition, the relatively low drain current and μ_h_ of DNTT on P3E1 may be attributed to its structural defects and crystallinity, and this issue will be discussed in the next section. With light illumination, all the EDLTs exhibited a boosted photocurrent, which is probably ascribed to the photoconductive effect of DNTT and the photogate effect of indigo carmine. We compared these results to the controlled experiments of the devices without indigo carmine, and the transfer characteristics are shown in Figure S15. All the devices exhibit 10^3^ times smaller current levels compared to those EDLTs with indigo carmine, which is due to the absence of anions that induce the hole channel. Thus, we suggest that the measured current may be attributed to the sum of the small drain current and the gate leakage current. The sensitivity (*S*) of the EDLT to light was defined as the ratio of (*I*_p_ – *I*_d_)/*I*_d_, where *I*_p_ is the photocurrent under light illumination and *I*_d_ is the dark current. Accordingly, Figure 2d presents the sensitivity of EDLTs to various BCP electrolytes. The maximal *S* is (9.7 ± 3.7) × 10^4^, (2.2 ± 1.2) × 10^4^, and (4.9 ± 5.8) × 10^2^ for P3E1, P1E1, and P1E7, respectively. Therefore, we can see that the BCP composition plays an important role in the EDL and photoresponding capability.

**Table 1:** Summary of the Device Parameters Measured in the Photonic Electrical Double-Layer Transistors Comprising the BCP/Indigo Carmine Electret and DNTT Channel

	P3E1	P1E1	P1E7
*C*_areal_ (nF cm^–2^)^a^	28.2 ± 3.3	60.3 ± 2.0	295.9 ± 11.1
μ_h_ (cm^2^ V^–1^ s^–1^)^b^	(6.76 ± 0.89) × 10^–4^	(2.61 ± 0.09) × 10^–3^	(7.15 ± 0.27) × 10^–3^
*I*_p_ (nA)^c^	0.474 ± 0.044	0.077 ± 0.005	0.092 ± 0.006
*t*_r_ (ms)^d^	869	575	575
*t*_f_ (ms)^d^	731	518	576
*R* (μA W^–^^1^)^e^	6.12 ± 0.57	0.99 ± 0.06	1.19 ± 0.08
EQE (%)^f^	(1.69 ± 0.16) × 10^–3^	(2.73 ± 0.17) × 10^–4^	(3.29 ± 0.22) × 10^–4^
*I*_n_ (A Hz^–0.5^)^g^	5.74 × 10^–14^	6.68 × 10^–14^	6.15 × 10^–14^
NEP (W Hz^–0.5^)^h^	(9.38 ± 0.87) × 10^–9^	(6.75 ± 0.41) × 10^–8^	(5.17 ± 0.35) × 10^–8^
D* (Jones)^i^	(2.38 ± 0.22) × 10^6^	(3.31 ± 0.20) × 10^5^	(4.33 ± 0.29) × 10^5^

a Areal capacitance of the BCP electrolyte measured from a metal–insulator–metal device structure.

b Hole mobility fitted from the saturation regime of transfer curves.

c Photocurrent of the device illuminated with 450 nm light under light intensity of 155 mW cm^–2^.

d Rise time and fall time derived from the transient photocurrent characteristics.

e Photoresponsivity calculated from *R* = *I*_p_/*P*_i_*A*, where *P*_i_ is the incident intensity of the 450 nm light and *A* is the channel area.

f EQE = (*R* × *h* × *c*)/(*e* × λ) with 450 nm light illumination.

g Dark current noise measured at 1 Hz.

h Noise equivalent power calculated from NEP = *I*_n_/*R*.

i Specific detectivity according to the 450 nm light illumination and the dark current noise measured at 1 Hz.

### Morphological Characterization of the DNTT Channel

**Figure 3:**
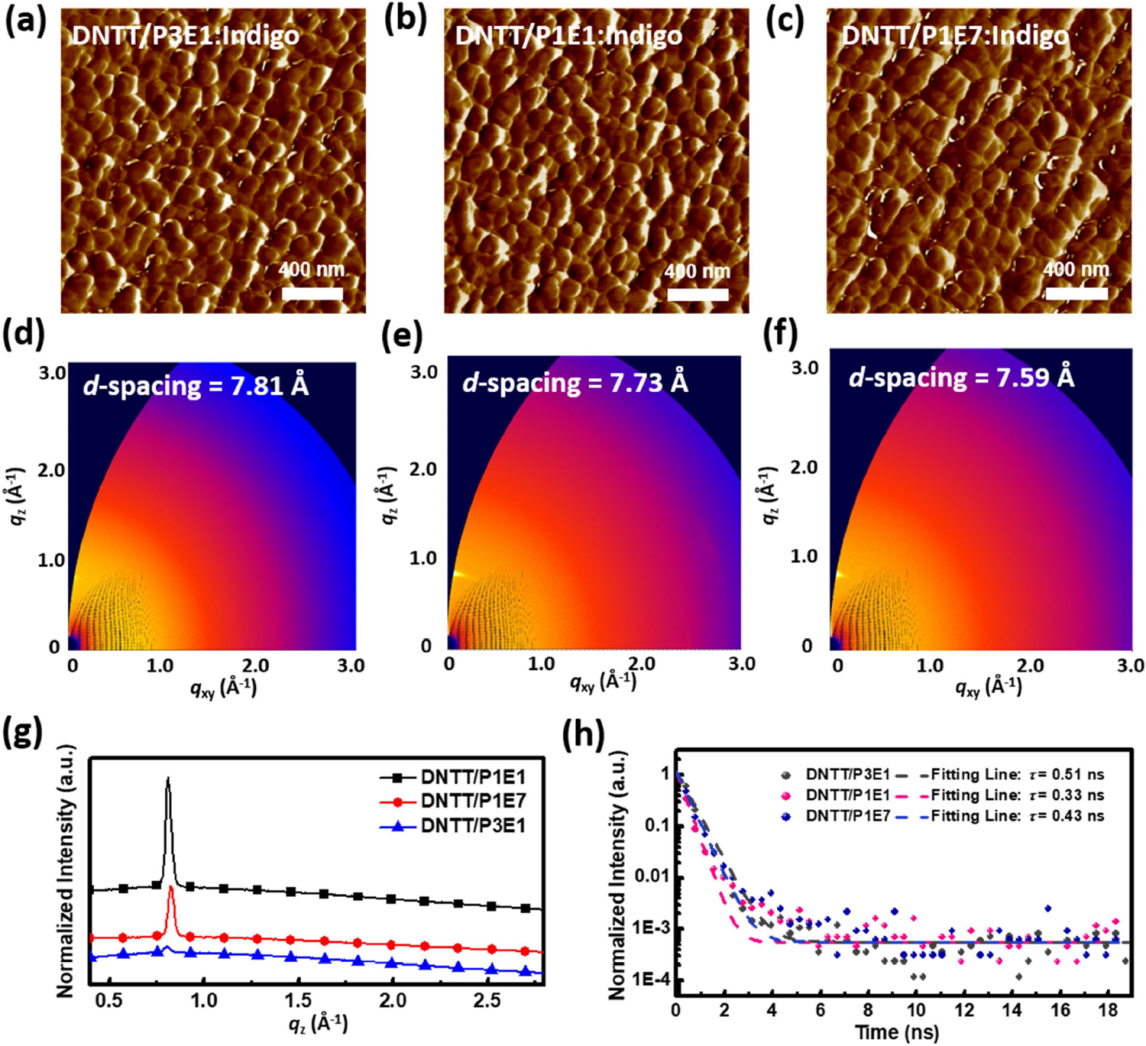
(a–c) AFM phase images, (d, f) 2D GIXD patterns of DNTT deposited on (a, d) P3E1, (b, e) P1E1, and (c, f) P1E7 with indigo carmine, and (g) corresponding 1D GIXD line-cutting profiles. (h) TRPL decay profiles of DNTT deposited on different BCP electrolytes.

To gain insight into the channel layer, the morphology and solid-state stacking of DNTT on these BCP electrolytes are investigated. Figure 3 presents the AFM phase images and GIXD patterns of DNTT. As can be seen, DNTT/P1E7 exhibited a larger grain size and a smaller *d*-spacing (7.59 Å) than that on P3E1 or P1E1, representing its densely packed and crystalline structure. This disparity is attributed to the most hydrophilic surface of P1E7:indigo carmine. Therefore, DNTT/P1E7 exhibited the highest dark current and mobility in dark conditions. In contrast, DNTT/P3E1 presented a small grain size and a large *d*-spacing (7.81 Å), indicating its loosely packed and less ordered structure. It is understood that the structural defect of semiconductors may contribute to charge trapping in transistors. Therefore, DNTT/P3E1 is more defect-rich than P1E1 and P1E7 and eventually enhances its high sensitivity to light and relatively low mobility. In addition, the TRPL technique was applied to gain insight into the exciton dynamics of DNTT on the BCP electrolytes. Figure 3h displays the TRPL decay profiles of the DNTT. A longer exciton lifetime (τ) is beneficial for forming separated free charges to boost the photocurrent of the device. Therefore, DNTT/P3E1, with a prolonged τ of 0.51 ns, in comparison to those of 0.33 and 0.43 ns for DNTT/P1E1 and DNTT/P1E7, exhibited the highest sensitivity to light. This outperformance may be attributed to the smaller grains and looser packing of DNTT on P3E1, so the exciton quenching is slightly mitigated.

### Photo-EDLT Characterization

**Figure 4:**
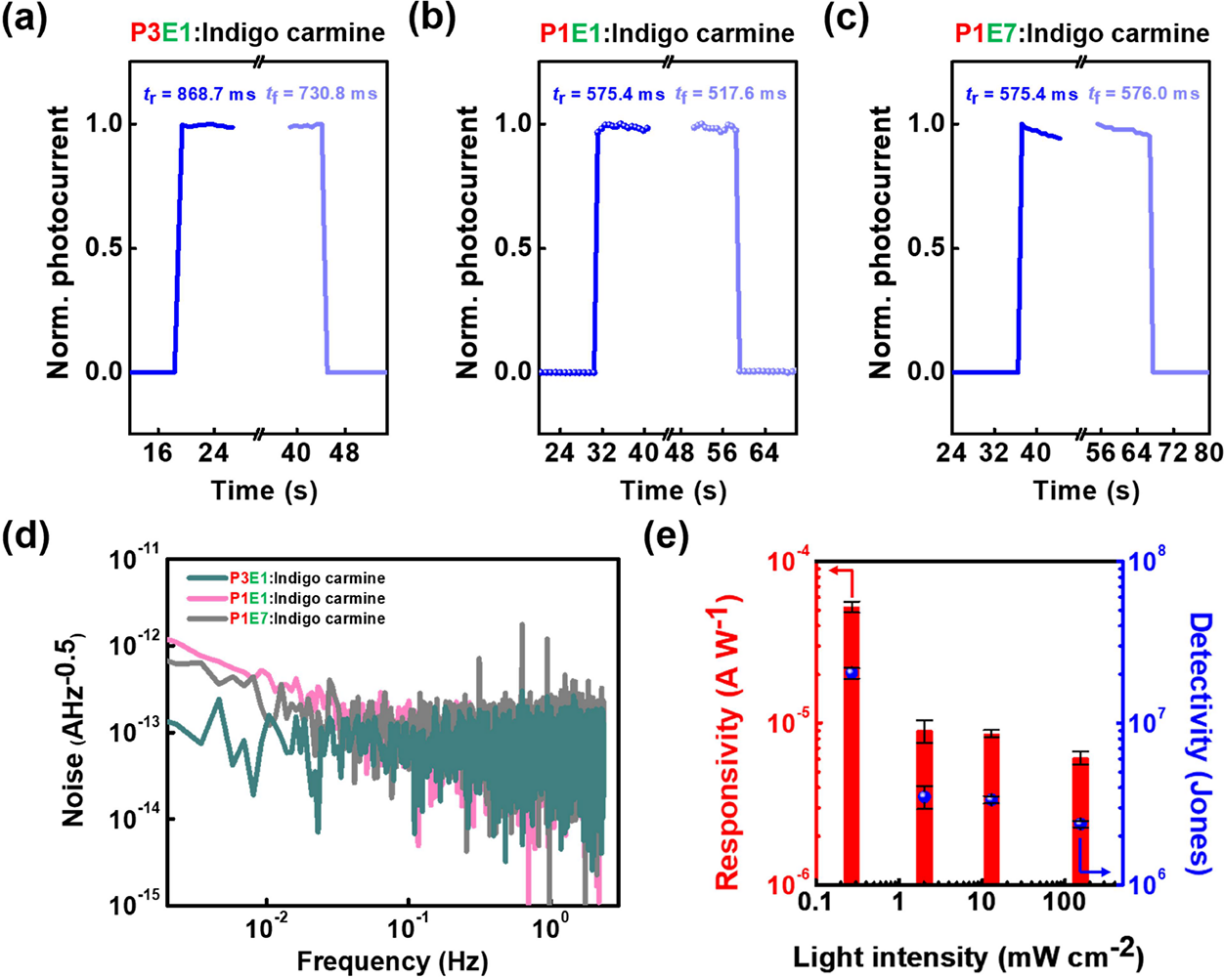
Transient photocurrent characteristics of the EDLT comprising BCP electrolytes comprising (a) P3E1, (b) P1E1, and (c) P1E7 with indigo carmine. Note that the drain voltage was fixed at −1 V, and the intensity of the 450 nm light was 34 mW cm^–2^. (d) Dark current noise of the EDLT devices. (e) Relationship between the photoresponsivity (*R*), specific detectivity (D*), and the intensity of 450 nm light for the EDLT device comprising P3E1:indigo carmine.

To investigate the photodetecting performance of EDLTs, the transient photocurrent characteristics under different light intensities were evaluated, and the testing condition was set to −1 V for drain voltage and with zero gate bias. Figure 4a–c depicts the photoresponse for each photo-EDLT, and the rise/fall time (*t*_r_/*t*_f_) was calculated through the time interval between 10 and 90% of the saturation photocurrent. Hence, the *t*_r_/*t*_f_ was derived as 869/731 for P3E1, 575/518 for P1E1, and 575/576 ms for P1E7, respectively. Although P3E1 had the highest sensitivity, its rising/falling responses took a longer time than its analogs to reach a steady state; and it can be ascribed to the impeded ionic motions of indigo carmine in the P4VP matrix that leads to the longest relaxation time. Figure S16 displays the transient photocurrent characteristics of the reference devices comprising P4VP or PEO with indigo carmine. As can be seen, the P4VP-based device showed a high photocurrent and a delayed relaxation; in contrast, the PEO-based device exhibited a low photocurrent and a fast response. Although P1E1 and P1E7 possessed higher contents of PEO leading to rapid rising/falling responses, the lack of surface charge traps may yield inferior photoresponses. Accordingly, Figure S17 shows the devices’ photoresponse to 450 nm light with a light intensity of 155 and 182 mW cm^–2^. The photoresponsivity (*R*) of EDLTs was calculated following eq 2 and summarized in Table 1:
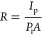
2where *P*_i_ is the power of the light source, and *A* is the channel area for light detection. The *R* values are (6.12 ± 0.57) × 10^–6^ A W^–^^1^ for P3E1, (0.99 ± 0.06) × 10^–6^ A W^–^^1^ for P1E1, and (1.19 ± 0.08) × 10^–6^ A W^–^^1^ for P1E7, respectively. Among all the photo-EDLTs, P3E1 with the optimized BCP composition had the highest photocurrent of (0.474 ± 0.044) × 10^–10^ A and the highest *R* of (6.12 ± 0.57) × 10^–6^ A W^–^^1^. The external quantum efficiency (EQE) was calculated based on the following equation: EQE = (*R* × *h* × *c*)/(*e* × λ), where *h* is the Planck constant, 6.63 × 10^–34^ m^2^ kg s^–1^; *c* is the speed of light, 3 × 10^8^ m s^–1^; *e* is the elementary charge, 1.6 × 10^–19^ C; and λ is the wavelength of incident light, 450 nm. The EQEs of P3E1, P1E1, and P1E7 were (1.69 ± 0.16) × 10^–3^ %, (2.73 ± 0.17) × 10^–3^ %, and (3.29 ± 0.22) × 10^–3^ %, respectively. Next, to understand the impact of morphology on photo-EDLTs, we fabricated the devices made by polymer blend electrolytes in which the blending weight ratios for P4VP and PEO were 3:1, 1:1, and 1:7. Figure S18 shows the transient photoresponse of the devices, and the *R* values are 1.83 × 10^–6^ A W^–^^1^ for 3:1, 1.80× 10^–7^ A W^–^^1^ for 1:1, and 1.12 × 10^–6^ A W^–^^1^ for 1:7. All three photo-EDLTs show declined *R*, which is due to severe aggregation of the parent polymers and irregular surface condition. In addition, to investigate the impact of salt loading on the device performance, reference devices comprising P3E1 with different contents of indigo carmine were fabricated and characterized. As can be seen in Figure S19, P3E1 with 10 wt % indigo carmine presented a similar performance to that observed in Figure 2a. By contrast, as the salt loading increased to 40 wt %, the photocurrent decreased and indicated that the excessive amount of indigo carmine was unfavorable for the photoresponse of EDLT.

Next, the dark current noise (*I*_n_) of the EDLTs was evaluated, and the results are shown in Figure 4d. The 1/f-type noise current of P3E1 is relatively lower than those of the other two counterparts, which is ascribed to its flattest surface roughness. The reduced contact area between DNTT and EDL can efficiently reduce the interfacial traps that result in fluctuation of the channel current and flicker noise. The *I*_n_ at 1 Hz for the EDLTs showed similar levels of 5.74 × 10^–14^ A Hz^–0.5^ for P3E1, 6.68 × 10^–14^ A Hz^–0.5^ for P1E1, and 6.15 × 10^–14^ A Hz^–0.5^ for P1E7. Subsequently, the noise equivalent power (NEP) and the specific detectivity (D*) were derived from the following eqs 3 and (4):
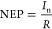
3
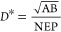
4where *B* equals one in one-hertz output bandwidth. The calculated NEP and D* are summarized in Table 1, and Figure S20 shows the transient response of P3E1 under different light intensities. The D* for P3E1, P1E1, and P1E7 are (2.38 ± 0.22) × 10^6^, (3.31 ± 0.20) × 10^5^, and (4.33 ± 0.29) × 10^5^ Jones, respectively. The highest detectivity observed for P3E1 originated from the strongest photoresponse. Notably, as the light intensity decreased from 155 to 0.27 mW cm^–2^, the D* of the P3E1-based device increased from (2.38 ± 0.10) × 10^6^ to (2.04 ± 0.16) × 10^7^ Jones (Figure 4e). Additionally, we compared the results when applying a drain current of −3 V. Figure S21a demonstrates the dark noise current, and Figure S21b shows the transient photoresponse when using 450 nm light with 155 mW cm^–2^ light intensity. The D* for P3E1, P1E1, and P1E7 are 3.52 × 10^6^, 1.10 × 10^5^, and 8.78 × 10^5^ Jones, respectively. Although higher drain voltage brings higher photocurrent, there is no obvious increase in D* due to the higher noise current under a drain voltage of −3 V. Therefore, we consider it more advantageous to apply a lower voltage for the low-power-consumption application.

**Figure 5:**
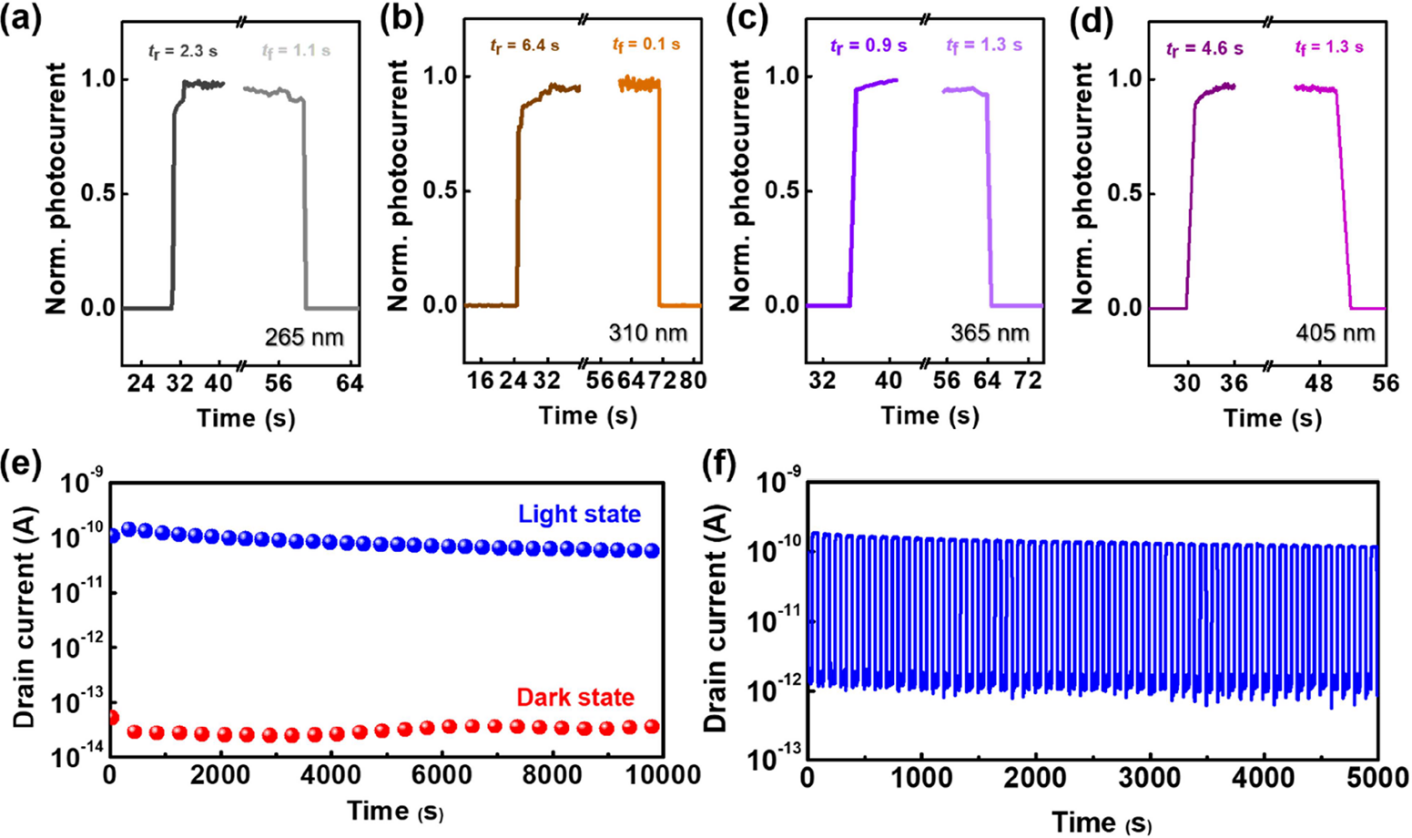
Transient photocurrent characteristics of the EDLT comprising P3E1 and indigo carmine with (a) 265, (b) 310, (c) 365, and (d) 405 nm light illuminations. Note that the drain voltage was fixed at −1 V, and the light intensity spanned the range 15–30 mW cm^–2^. (e) Long-term stability and (f) switchability characteristics of the EDLT comprising P3E1:indigo carmine under 450 nm light illumination (34 mW cm^–2^) or in a dark state.

In addition to the detection of 450 nm light, the photo-EDLT comprising P3E1 was applied to detect different wavelengths of light, including the ultraviolet C (UVC; 265 nm; Figure 5a), ultraviolet B (UVB; 310 nm; Figure 5b), ultraviolet A (UVA; 365 nm; Figure 5c), and purple (405 nm; Figure 5d) light. The drain voltage was fixed at −1 V, and the light intensity spanned the range of 15–30 mW cm^–2^. As can be seen, the photo-EDLT presented similar sensing capabilities with a sensitivity of 347, 232, 2454, and 418 to UVC, UVB, UVA, and purple light. The results indicated that both the indigo carmine and DNTT contributed to the enhancement in photocurrent, and the photo-EDLT developed can be used in a broad range of light. Next, the long-term operation (Figure 5e), switching test (Figure 5f), and ambient stability (Figure S22) of the photo-EDLT comprising P3E1 were finally evaluated. The illumination was conducted with 450 nm light (34 mW cm^–2^) along 10,000 s or 50 consecutive cycles for the long-term operation and switching test. As can be seen, the photo-EDLT with BCP electrolyte exhibited moderate ambient stability, decent long-term stability, and switchability with a high current contrast of 10^5^ between the light and dark states. In addition, the photo-EDLT comprising P3E1 presented ultralow power consumptions of 0.59 nW under 450 nm light illumination and 0.32 pW under dark state conditions are much lower than other reported photo-EDLT with 0.2–0.6 μW under light state and approximately 1 nW under dark state.^45,46^

**Figure 6:**
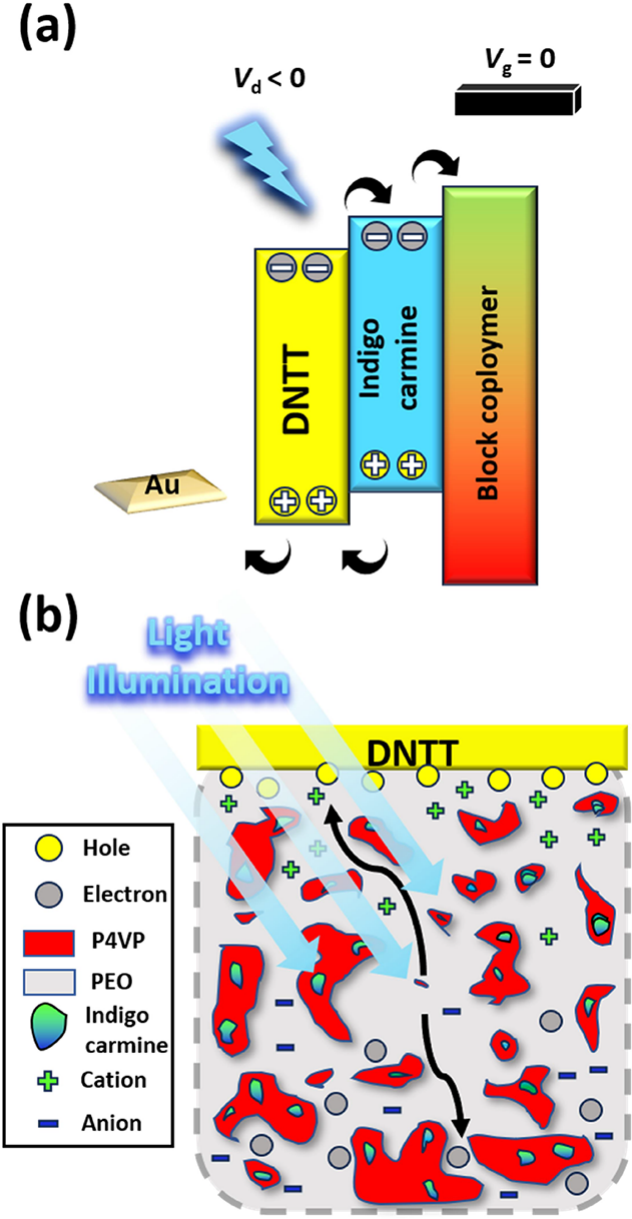
Schematic illustrations of (a) device working mechanism and (b) relationship between the composition of BCP electrolytes and device performance of photo-EDLT.

To explain the decent performance of photo-EDLT comprising the BCP electrolyte, the contribution of each component in the device is respectively discussed. Figure 6a presents the device’s working mechanism under light illumination, and Figure 6b portrays the relationship between the composition of BCP electrolytes and the device performance of photo-EDLT. With regard to the BCP structure, P4VP serves as the interfacial charge trap, and PEO is responsible for ion conduction. PEO is a highly ion-conductive polymer with high crystallinity. P4VP is therefore selected as the other block due to the weak lone-pair interaction of pyridine with ions and the charge-trapping capability, making it suitable to combine with PEO to formulate the BCP electrolyte in EDLT. The hydrophilic nature of PEO results in the polymer tending to move toward the environment, leading the P4VP to assemble while being surrounded by the PEO block. Regarding the photoresponse, both indigo carmine and DNTT generated excitons, and the excitons can separate into charges to boost the photocurrent of the device. Notably, the photogenerated hole was transported through the DNTT channel. At the same time, the electron remained at the interface to form a built-in electric field to boost the carrier concentration inside the channel further. With light illumination, the photo-EDLTs exhibited a boosted photocurrent, which is probably ascribed to the photoconductive effect of DNTT and the photogate effect of indigo carmine.

The disparity in charge transport and photodetecting capability among the photo-EDLT developed with varied BCP compositions can be attributed to the EDL capacitance and the distribution of P4VP/PEO blocks at the contact interface with DNTT. Therefore, the device performance can be optimized by balancing the P4VP and PEO blocks in the BCP composition. Brixi et al. reported that the thin-film self-assembly of BCP electrolytes with an optimal block ratio can improve the performance of electrolyte-gated transistors.^47^ Therefore, the effects of block ratios and molecular weights of BCPs on the device performance were further investigated by introducing an additional BCP comprising P4VP_5k_-*b*-PEO_20k_ (P4E1) and compared with those of P4VP_16k_-*b*-PEO_5k_ (P3E1), P4VP_17k_-*b*-PEO_17k_ (P1E1), and P4VP_4.5k_-*b*-PEO_32k_ (P1E7). Figure S23 presents the device characteristics of P4E1, and the results indicate that P4E1 showed a comparable *t*_r_/*t*_f_, a slightly higher *R*, and a lower D* than did P3E1. Therefore, the optimal point of the block ratio lies approximately in this region. In addition, the molecular weight of the P4VP block in P4E1 is comparable to P1E7. We found that the performance of P4E1 is quite similar to P3E1 instead of P1E7. In addition, P3E1 also shares a similar P4VP molecular weight with P1E1, and the results again indicate the impact of the block ratio on the BCP electrolytes’ morphology and EDLT’s performance. Therefore, we postulate that the effect of the block ratio is more important than the individual molecular weight in each block. Even though P3E1 presented a slightly longer response time than its analogs with higher PEO contents, it demonstrated the best photodetecting capability owing to the abundant surface traps induced by immobile indigo carmine in the P4VP matrix that facilitate exciton dissociation at the semiconductor/dielectric interface.

## Conclusions

A series of BCP electrolytes comprising P4VP-*b*-PEO with varied block ratios and indigo carmine salt were applied in EDLT to develop ultralow-power-consumption photodetectors. Based on the experimental results, we found that P4VP serves as the interfacial charge trap and PEO is responsible for ion conduction in the BCP electrolyte. Both indigo carmine and DNTT absorb light to generate excitons, and the photogenerated excitons can separate into charges to boost the photocurrent of the device. The disparity in charge transport and photodetecting capability of the photo-EDLT with varied BCP electrolytes can be attributed to the EDL capacitance and the distribution of P4VP/PEO blocks at the contact interface with DNTT. Therefore, the device performance can be optimized by striking a balance between the P4VP and PEO blocks in the BCP composition. Accordingly, EDLT comprising P3E1 and indigo carmine provides the highest D* of 2.1 × 10^7^ Jones, with ultralow power consumptions of 0.59 nW under 450 nm light illumination and 0.32 pW under a dark state. The results of this study provide an initial concept of photoactive BCP electrolytes for EDLT to develop low-power-consumption photodetectors. In addition, the ion migration of the indigo carmine determines the dynamic properties of the photo-EDLT, and there is an inevitable trade-off between the photoresponsivity and the response time. Therefore, the selection of materials that can maintain high photocurrent without sacrificing response time is an upcoming challenge to be overcome.

## Supporting Information Available

Characteristics of the phototransistors; water contact angles of the block copolymer electrolyte films; FTIR characterizations of the thin films; SEM and GISAXS morphologies of the thin films; and electrochemical characterization of the constituent materials (PDF)
