# Supplementary material for: Electrical Double-Layer Transistors Comprising Block Copolymer Electrolytes for Low-Power-Consumption Photodetectors
Source: ACS Appl Mater Interfaces. 2024 May 6;16(19):25042–52. doi: 10.1021/acsami.4c01959 (PMC11103659; doi:10.1021/acsami.4c01959)
Supplement: Supplementary file 1 — am4c01959_si_001.pdf [file am4c01959_si_001.pdf]

Supporting Information

Electrical Double-Layer Transistors Comprising Block Copolymer Electrolytes for Low-Power-Consumption Photodetectors

Hung-An Lin,[a] *Yi-Hsun Weng,*[b] *Tiffany Mulia,*[b] *Cheng-Liang Liu,*[c,d]

Yan-Cheng Lin,[c,e*] *Yang-Yen Yu,*[a*] *and Wen-Chang Chen*[a,c,*]

[a] Department of Materials Engineering, Ming Chi University of Technology, New Taipei City 24301, Taiwan.

[b] Department of Chemical Engineering, National Taiwan University, Taipei 10617, Taiwan

[c] Advanced Research Center for Green Materials Science and Technology, National Taiwan University, Taipei 10617, Taiwan

[d] Department of Materials Science and Engineering, National Taiwan University, Taipei 10617, Taiwan

[e] Department of Chemical Engineering, National Cheng Kung University, Tainan 70101, Taiwan

[*]Corresponding author. E-mail: ycl@gs.ncku.edu.tw (Y.-C. Lin); yyyu@mail.mcut.edu.tw (Y.-Y. Yu); chenwc@ntu.edu.tw (W.-C. Chen)

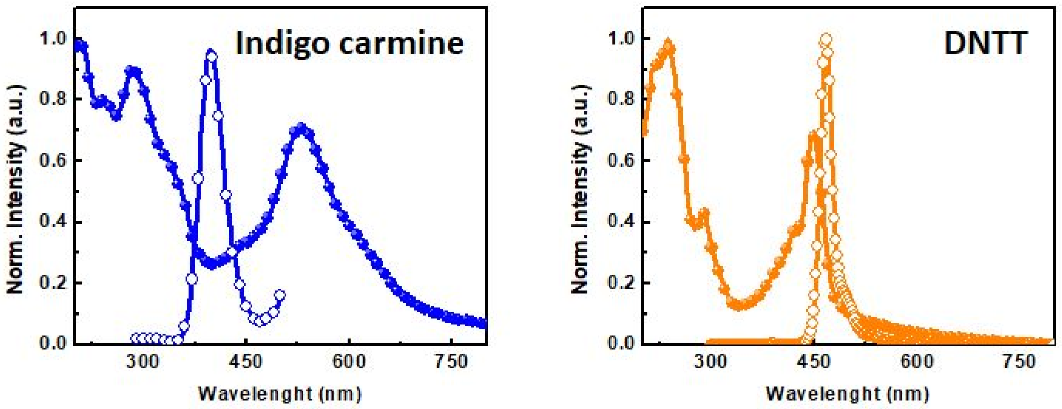

Figure S1. UV-vis absorption (solid symbol) and PL emission (open symbol) spectra of indigo carmine (left; λ_{ext} = 300 nm) and DNTT (right; λ_{ext} = 450 nm).

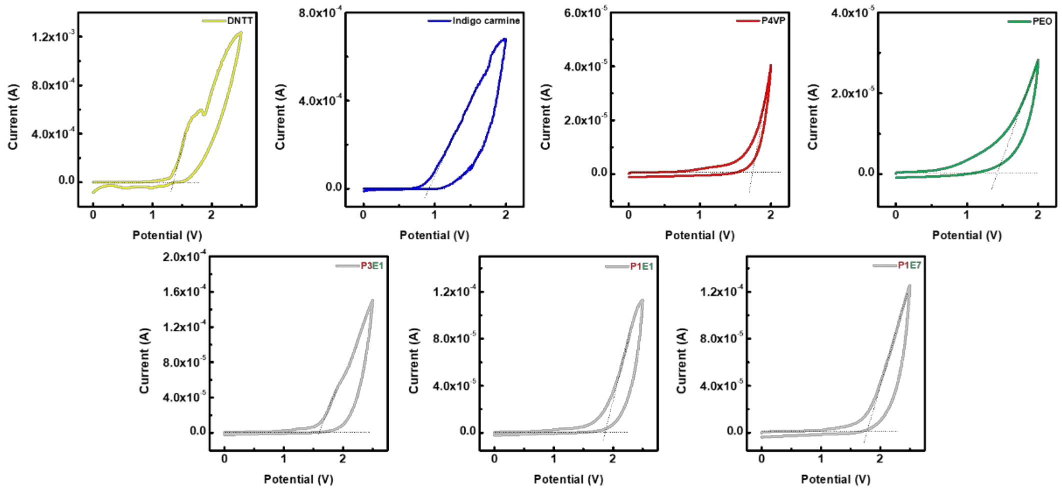

Figure S2. CV profiles of DNTT, indigo carmine, P4VP, PEO, P3E1, P1E1, and P1E7. Note that the measurement was conducted in a three-electrodes system with a platinum auxiliary electrode and an Ag/AgCl reference electrode, and the sweeping rate was fixed at 100 mV s^{-1}.

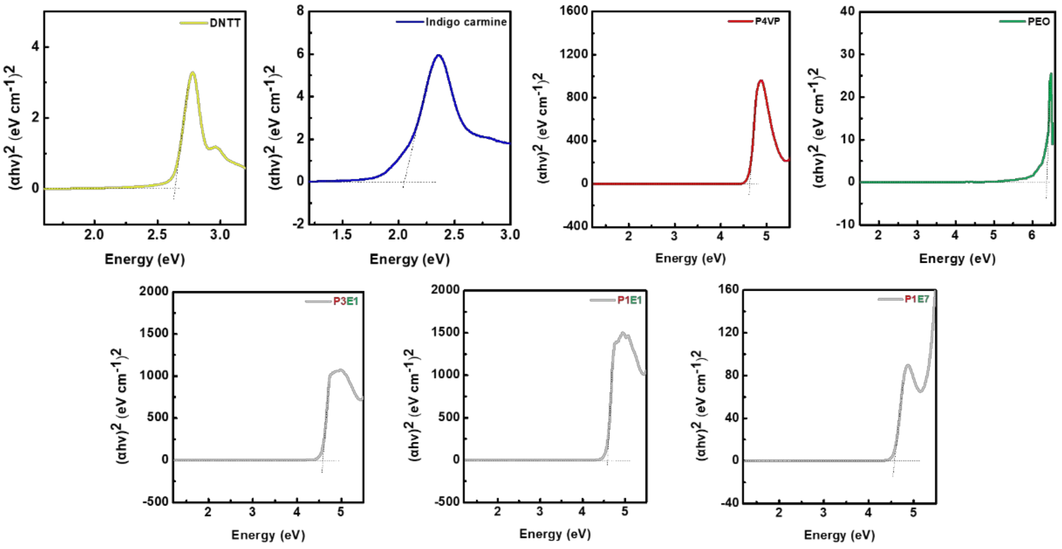

Figure S3. Tauc plots of DNTT, indigo carmine, P4VP, PEO, P3E1, P1E1, and P1E7.

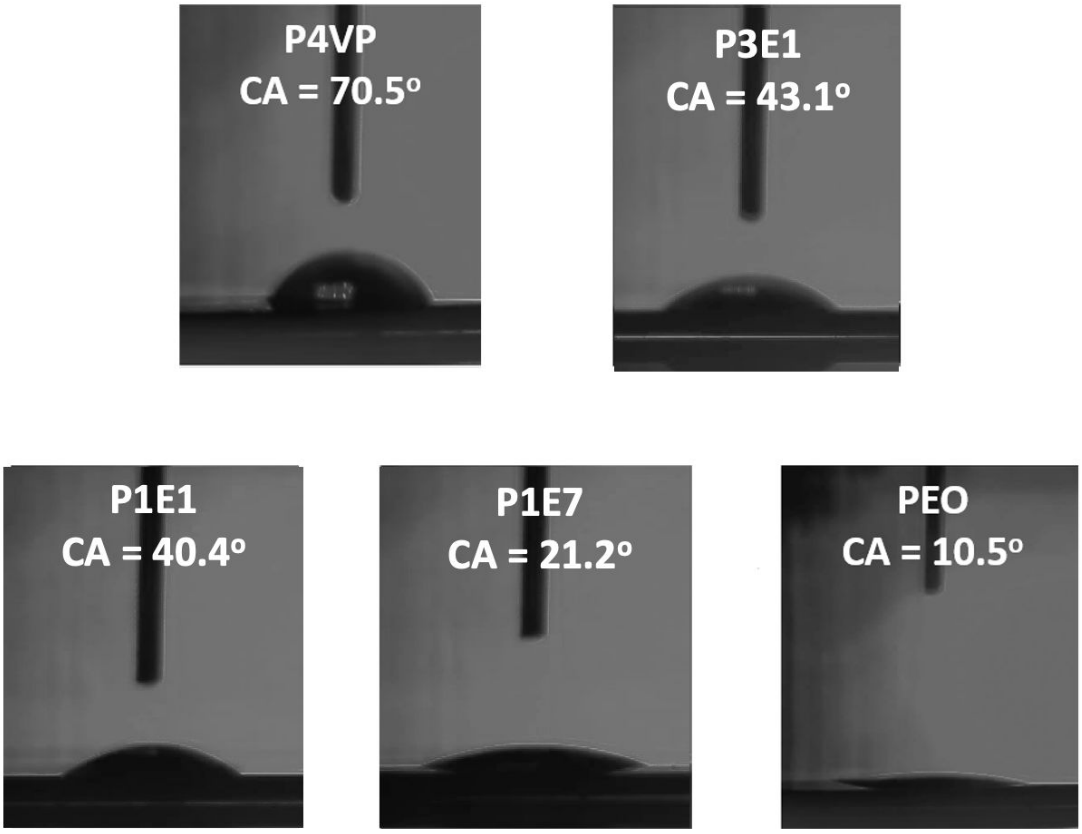

Figure S4. Water contact angle (CA) of the block copolymer polyelectrolyte films and their analogs of P4VP and PEO with indigo carmine.

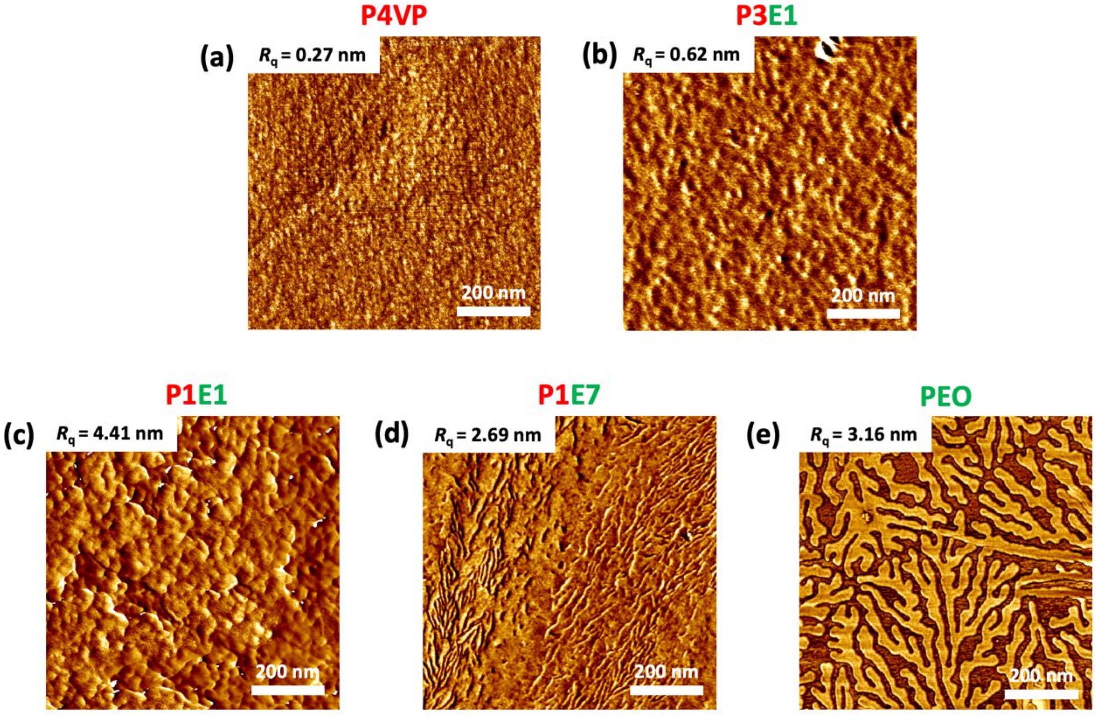

Figure S5. AFM topographies of the polymer films consisting of (a) P4VP, (b) P3E1, (c) P1E1, (d) P1E7, and (e) PEO.

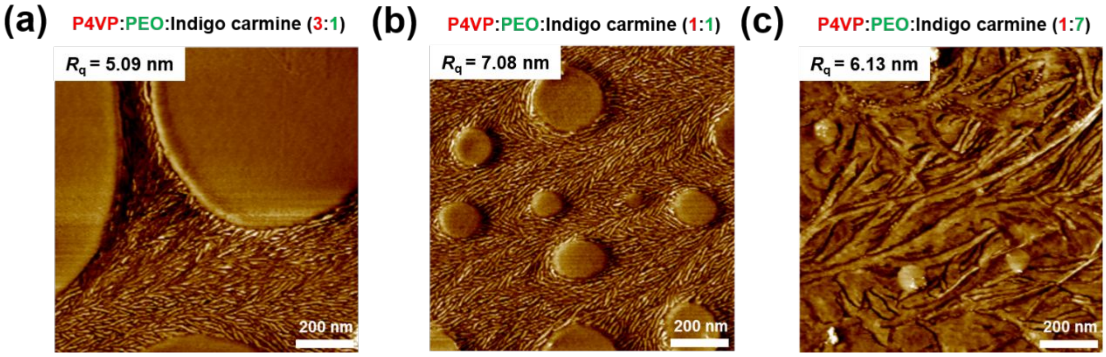

Figure S6. AFM topographies of the polymer blend films consisting of (a) P4VP:PEO (3:1), (b) P4VP:PEO (1:1), and (c) P4VP:PEO (1:7) with indigo carmine.

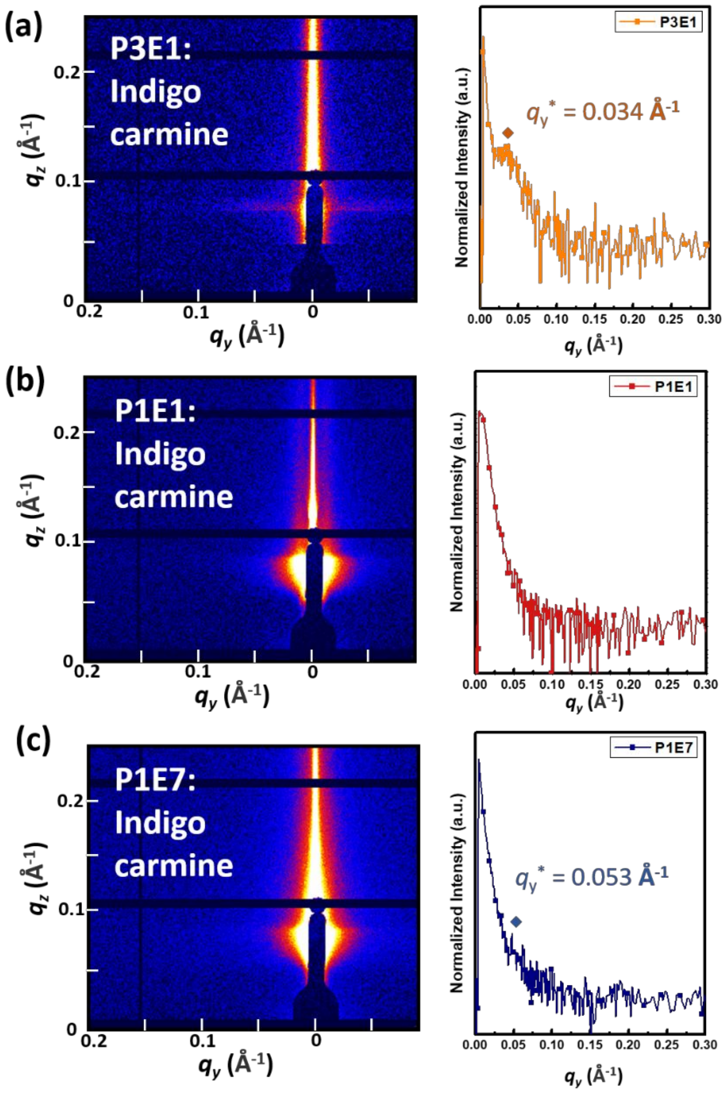

Figure S7. GISAXS 2D patterns (left) and 1D line-cutting profiles (right) of the BCP electrolyte films comprising (a) P3E1, (b) P1E1, and (c) P1E7 with indigo carmine.

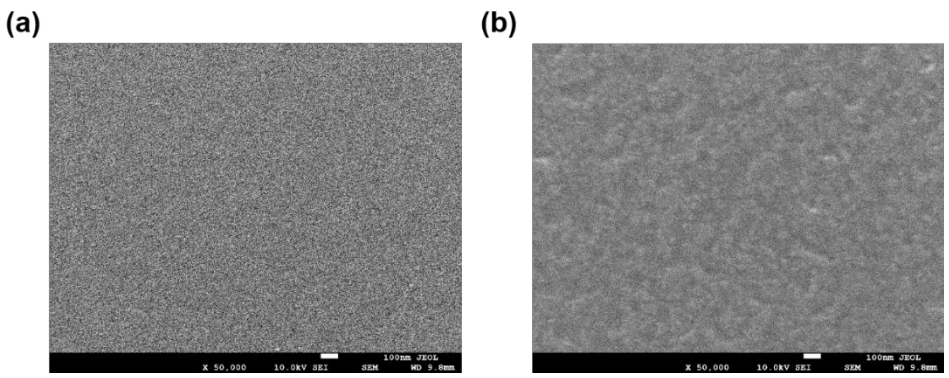

Figure S8. SEM images of (a) P3E1:indigo carmine and (b) P1E1:indigo carmine.

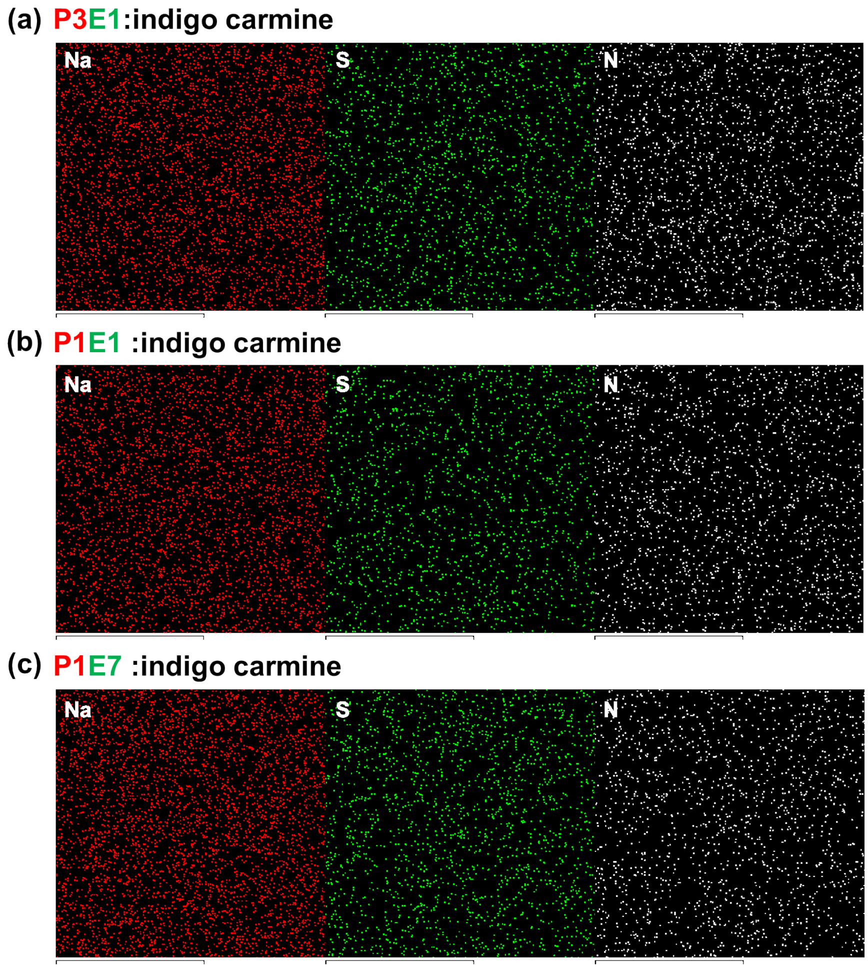

Figure S9. EDX element mapping of (a) P3E1:indigo carmine, (b) P1E1:indigo carmine, and (c) P1E7:indigo carmine.

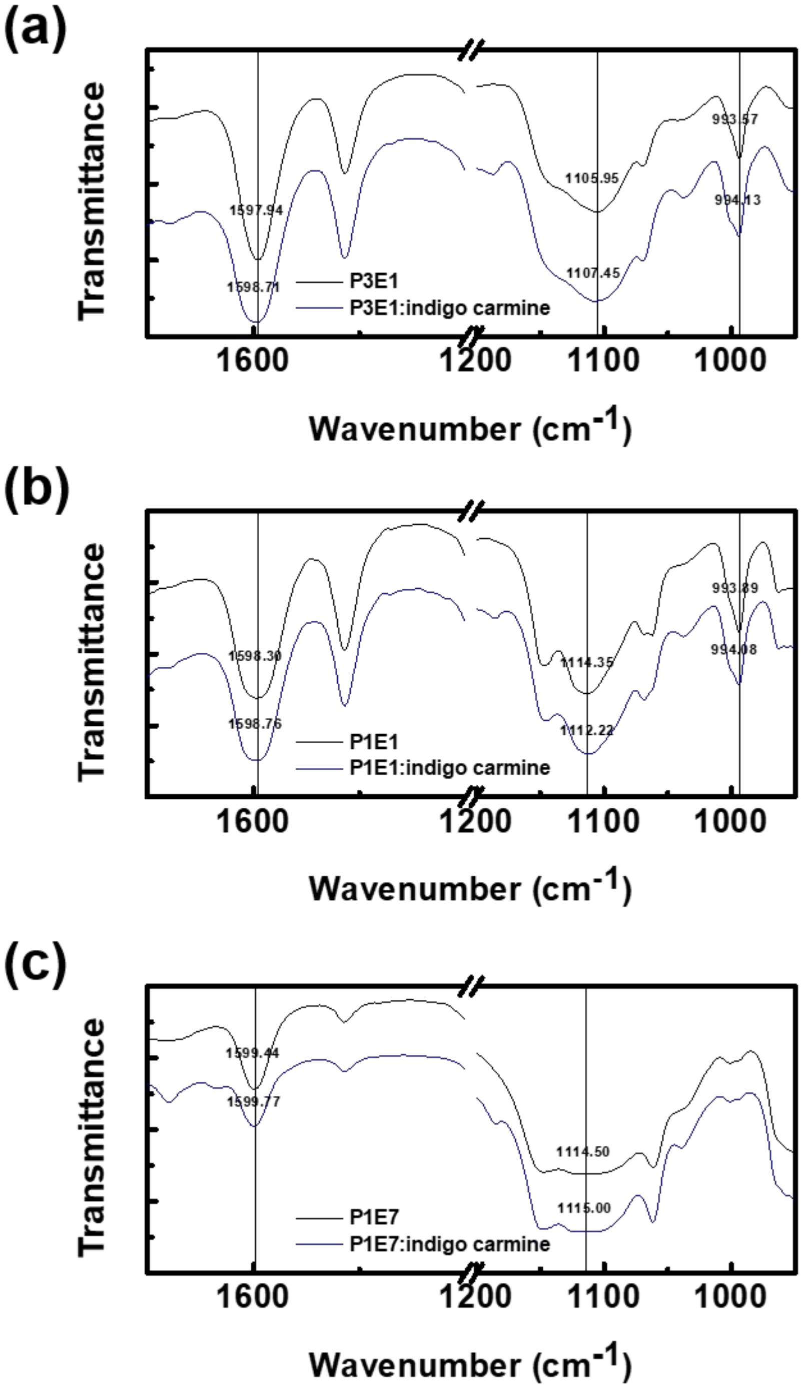

Figure S10. FTIR spectra of (a) P3E1 and P3E1:indigo carmine, (b) P1E1 and P1E1:indigo carmine, and (c) P1E7 and P1E7:indigo carmine.

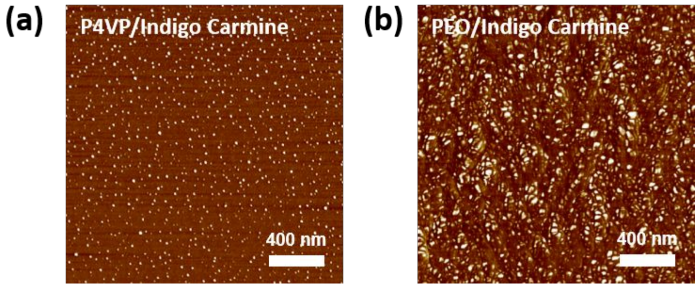

Figure S11. AFM phase images of (a) P4VP and (b) PEO homopolymers with 20 wt% of indigo carmine in comparison to the polymers. The results indicate an inhomogeneous allocation of indigo carmine inside the polymer matrix.

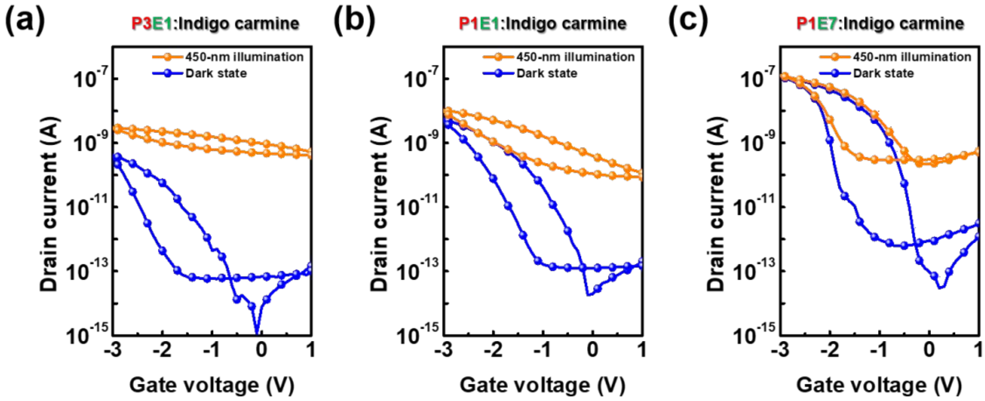

Figure S12. Hysteresis of the EDLT comprising (a) P3E1, (b) P1E1, and (c) P1E7 with indigo carmine in the dark state or under 450-nm light illumination. Note that the gate voltage applied was swept forward from 1 to –3 V and backward from –3 to 1 V. The drain voltage was fixed at –1 V and the light intensity was 155 mW cm^{-2}.

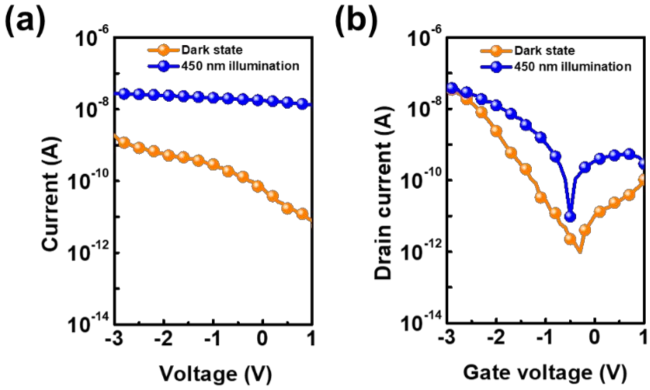

Figure S13. Transfer characteristics of the EDLT comprising (a) P4VP:indigo carmine and (b) PEO:indigo carmine in the dark state or under 450-nm light illumination. Note that the gate voltage applied in transfer curves was swept from 1 to –3 V, the drain voltage was fixed at –1 V, and the light intensity was 34 mW cm^{-2}.

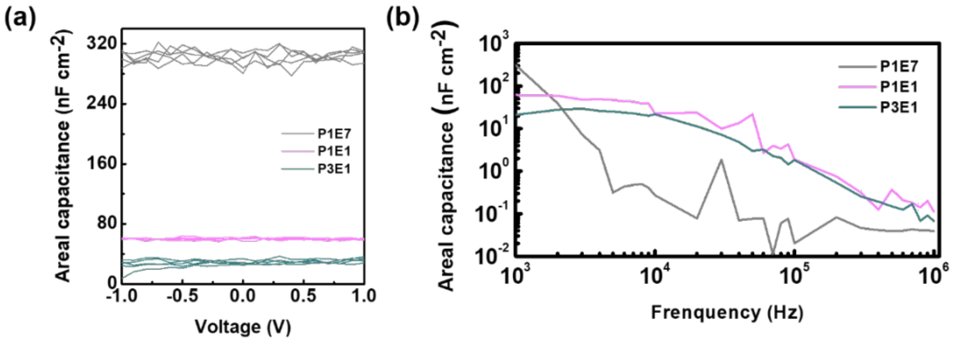

Figure S14. The C_{areal} of the EDL capacitors (a) under voltage of 1 V to −1 V, and (b) under frequencies from 1 kHz to 1 MHz.

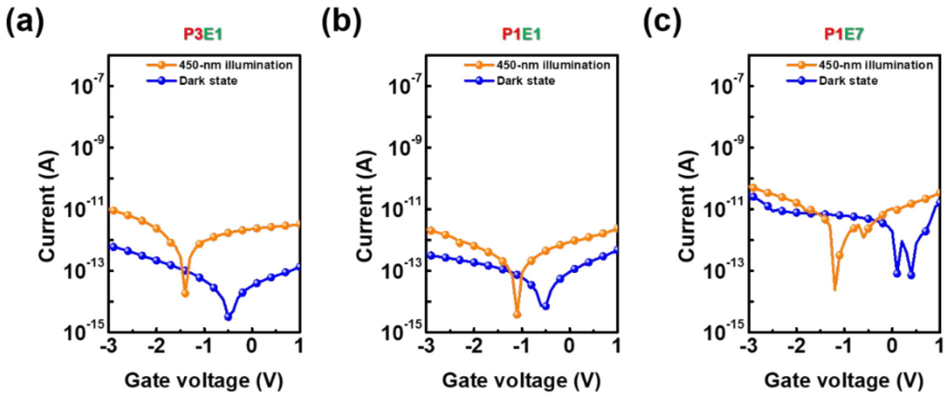

Figure S15. Transfer characteristics of the EDLT comprising (a) P3E1, (b) P1E1, and (c) P1E7 without indigo carmine in the dark state or under 450-nm light illumination.

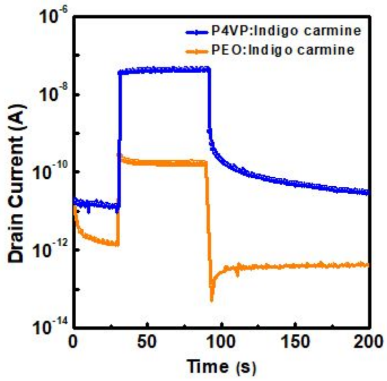

Figure S16. Transient photocurrent characteristics of the reference EDLT comprising polyelectrolytes with P4VP or PEO. Note that the drain voltage was fixed at –1 V, and the intensity of 450-nm light applied within 30–90 s was 155 mW cm^{-2}.

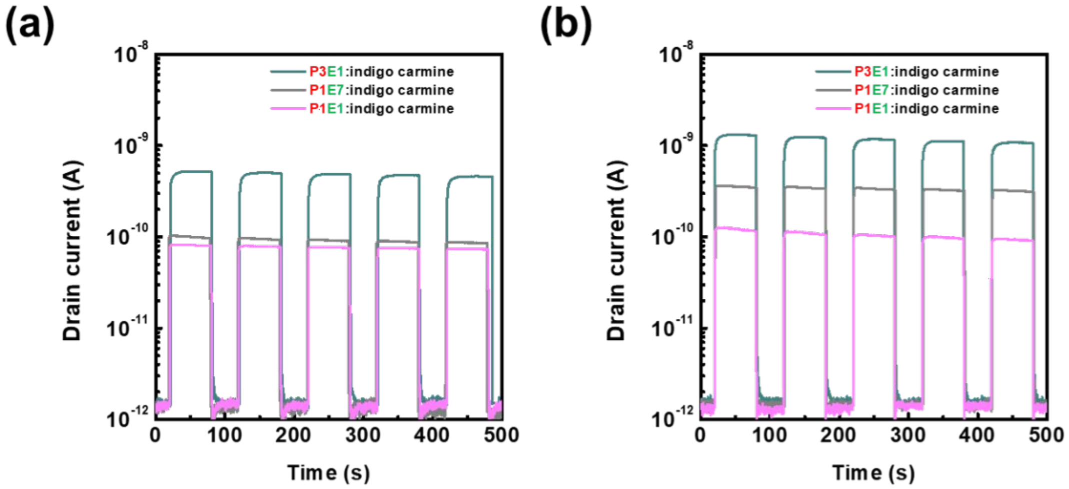

Figure S17. Transient photocurrent characteristics of the EDLT comprising BCP electrolytes with varied compositions. Note that the drain voltage was fixed at –1 V, and the intensity of 450-nm light was (a) 155 mW cm^{-2} and (b) 182 mW cm^{-2}.

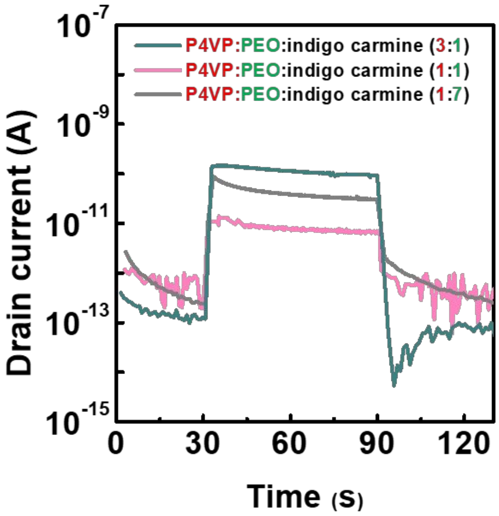

Figure S18. Transient photocurrent characteristics of the EDLT comprising polymer blend electrolytes with varied compositions. Note that the drain voltage was fixed at –1 V, and the intensity of 450-nm light applied within 30–90 s was 155 mW cm^{-2}.

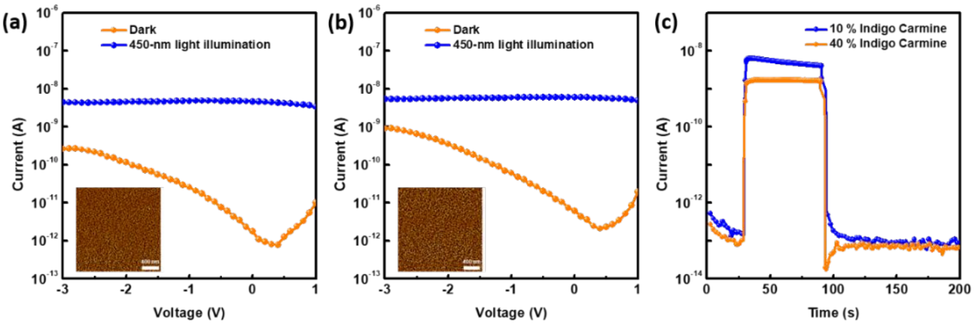

Figure S19. Transfer characteristics of the EDLT comprising P3E1 and (a) 10 wt% or (b) 40 wt% indigo carmine in the dark state or under 450-nm light illumination. (c) Transient photoresponse of BCP electrolytes under light illumination. Note that the gate voltage applied was swept from 1 to –3 V, the drain voltage was fixed at –1 V, and the light intensity was 34 mW cm^{-2}. The inset images in (a) and (b) are their AFM phase images.

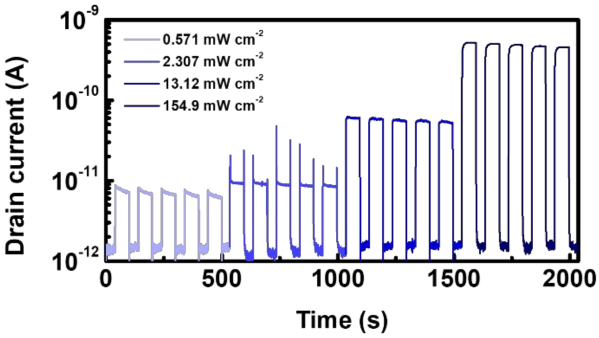

Figure S20. Transient photocurrent characteristics of P3E1 under different light intensities. Note that the drain voltage was fixed at –1 V.

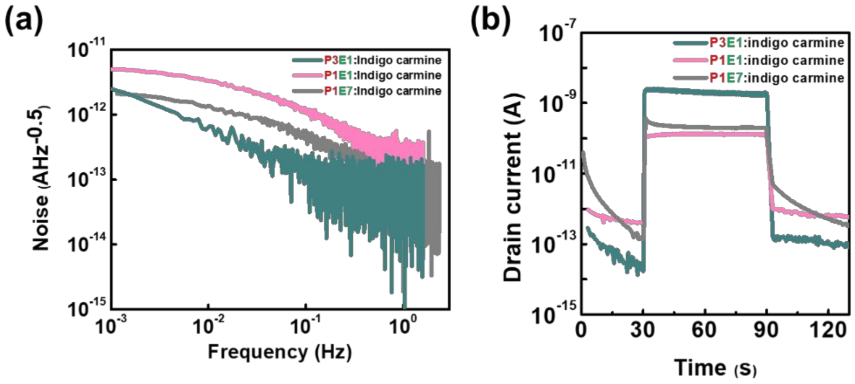

Figure S21. (a) Dark current noise of the EDLT devices. **(b)** Transient photocurrent characteristics of the EDLT comprising BCP electrolytes with varied compositions. Note that the drain voltage was fixed at –3 V, and the intensity of 450-nm light applied within 30–90 s was 155 mW cm^{-2}.

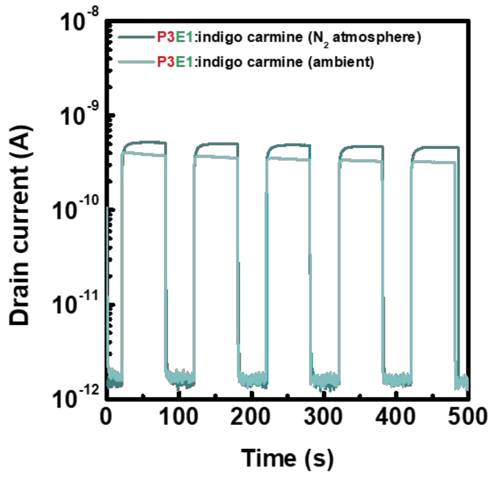

Figure S22. Transient photocurrent characteristics of P3E1 stored in an N_2 atmosphere or an ambient environment for a week. Note that the drain voltage was fixed at –1 V, and the intensity of 450-nm light was 155 mW cm^{-2}.

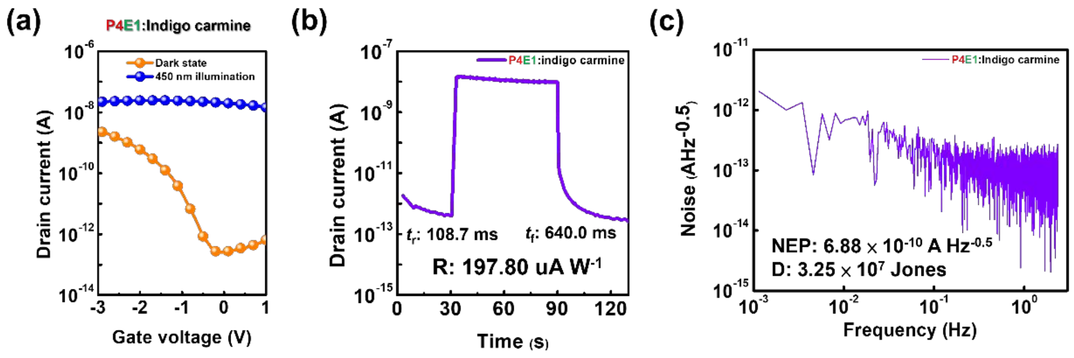

Figure S23. Comparison study of BCP's molecular weight and block ratio and the device performance: (a) transfer curves, (b) transient photocurrent characteristics, and (c) dark current noise of P4E1:indigo carmine. Note that the drain voltage was fixed at –1 V, and the intensity of 450-nm light applied was 155 mW cm^{-2}.
